# Allelopathic potential in rice - a biochemical tool for plant defence against weeds

**DOI:** 10.3389/fpls.2022.1072723

**Published:** 2022-12-14

**Authors:** Ferdoushi Rahaman, Abdul Shukor Juraimi, Mohd Y. Rafii, Kamal Uddin, Lutful Hassan, Abul Kashem Chowdhury, Sarker Mohammad Rezaul Karim, Bashir Yusuf Rini, Oladosu Yusuff, H. M. Khairul Bashar, Akbar Hossain

**Affiliations:** ^1^ Department of Crop Science, Faculty of Agriculture, University Putra Malaysia (UPM), Serdang, Malaysia; ^2^ Institute of Tropical Agriculture and Food Security, Universiti Putra Malaysia, Selangor, Malaysia; ^3^ Department of Land Management, University Putra Malaysia (UPM), Serdang, Malaysia; ^4^ Department of Genetics and Plant Breeding, Faculty of Agriculture, Bangladesh Agricultural University, Mymensingh, Bangladesh; ^5^ Department of Genetics and Plant Breeding, Faculty of Agriculture, Patuakhali Science and Technology University, Dumki, Patuakhali, Bangladesh; ^6^ Department of Biological Sciences, Federal University Gusau, Gusau, Nigeria; ^7^ On-Farm Research Division (OFRD), Bangladesh Agricultural Research Institute, Gazipur, Bangladesh; ^8^ Soil Science Division, Bangladesh Wheat and Maize Research Institute, Dinajpur, Bangladesh

**Keywords:** allelopathy, rice allelochemicals, eco-friendly weed management, mode of action, factors affecting allelochemicals, breeding for allelopathic traits

## Abstract

Rice is a key crop for meeting the global food demand and ensuring food security. However, the crop has been facing great problems to combat the weed problem. Synthetic herbicides pose a severe threat to the long-term viability of agricultural output, agroecosystems, and human health. Allelochemicals, secondary metabolites of allelopathic plants, are a powerful tool for biological and eco-friendly weed management. The dynamics of weed species in various situations are determined by crop allelopathy. Phenolics and momilactones are the most common allelochemicals responsible for herbicidal effects in rice. The dispersion of allelochemicals is influenced not only by crop variety but also by climatic conditions. The most volatile chemicals, such as terpenoids, are usually emitted by crop plants in drought-stricken areas whereas the plants in humid zones release phytotoxins that are hydrophilic in nature, including phenolics, flavonoids, and alkaloids. The allelochemicals can disrupt the biochemical and physiological processes in weeds causing them to die finally. This study insight into the concepts of allelopathy and allelochemicals, types of allelochemicals, techniques of investigating allelopathic potential in rice, modes of action of allelochemicals, pathways of allelochemical production in plants, biosynthesis of allelochemicals in rice, factors influencing the production of allelochemicals in plants, genetical manipulation through breeding to develop allelopathic traits in rice, the significance of rice allelopathy in sustainable agriculture, etc. Understanding these biological phenomena may thus aid in the development of new and novel weed-control tactics while allowing farmers to manage weeds in an environmentally friendly manner.

## 1 Introduction

Rice is still the most significant food crop on the planet, which is grown in a wide range of ecological conditions (Gaballah et al., 2021). Rice is one of the most economically significant staple food crops, especially in Asia, where most rice is produced and consumed (Aravind et al., 2015). Paddy rice is important to the country’s economy since it ensures food security, produces rural jobs and generates export earnings (Le, 2016; Xu et al., 2021). In the whole history of agriculture, the last fifty years have seen the highest levels of productivity. Because of the “Green Revolution,” which was made possible by advancements in agricultural science and technology, an estimated 1 billion people have been spared the misery of hunger and even starvation (Xu, 2010). However, weeds are the most serious biotic stresses (Rahaman et al., 2021), which typically outweigh the damages caused by any other kind of agricultural pest, including rodents, insects, diseases, and so on (Abouziena & Haggag, 2016). Indeed, the application of chemicals in controlling these bio-constraints leads to develop herbicide resistance and environmental pollution. Integration of crop allelopathy with existing agronomic practices can provide a more sustainable and novel approach to agricultural production methods (Li et al., 2020).

Allelopathic rice varieties produce and discharge allelochemicals that limit weed development and establishment, and might be included in a component of a combined weed-managing approach (Serra Serra et al., 2021). Allelopathy is an ecological phenomenon that occurs when plants, microbes, allelochemicals, and the environment interact (Abbas et al., 2021). Allelopathy occurs when a plant emits allelochemicals into the agroecosystem that interfere with neighbouring plants’ physiology, germination of seeds, typical growth and development, and longevity (Das et al., 2021). Allelochemicals, the rhizosphere microbiome, and their bio interaction are the heart of this natural weed-control strategy. Allelochemicals have enormous potential to manufacture biocontrol products. Thanks to the search for innovative active ingredients and the development of new manufacturing techniques brought about by the advent of biotechnology (Chaïb et al., 2021). Biological management in agriculture through the use of naturally occurring allelopathic substances has thus become essential and useful. Allelochemicals can be employed as useful techniques for increasing agricultural yields through sustainable weed management (Chou, 1999).

Understanding the link between allelochemicals and certain bacteria might assist to speed up the application of allelopathic qualities in farming. The effects of allelopathy depend on both species and concentration. However, the edaphic environment’s accumulation of allelochemicals affects soil chemistry (Lal et al., 2021). Agricultural crop seed germination, stand establishment, growth, yield, and physiology are all affected by the allelochemicals emitted by weeds (Zohaib et al., 2016). However, the allelochemicals in rice are a helpful tool to defend the growth and development of different weed species in the crop. Momilactones in rice, benzoxanoids in the rye, tabanone in cogongrass, alkaloids and flavonoids in fescue, anthratectone and naphthotectone in the teak (*Tectona grandis*), sorgoleone in sorghum, caffeic acid and calorigenic acids in sunflower, and abscisic acid beta-d-glucopyranosy have all been found as allelochemicals implicated in weed control (Ferguson et al., 2013). Eventually, plant allelopathy is often a win-win technique for managing weeds in the fields because it is an ecologically sound and resource-saving practice (Li et al., 2020). The microbial decomposition of rice husks produces hazardous chemicals such as p-coumaric acid, p-hydroxybenzoic acid, syringic acid, vanillic acid, ferulic acid, and o-hydroxy phenylacetic acid (Amb and Ahluwalia, 2016). Eventually, allelopathic rice cultivars may create and release allelochemicals that prevent paddy weeds from growing and establishing, making them an effective part of integrated weed control (Serra Serra et al., 2021). This review article exclusively focuses on different aspects of allelopathy especially in rice to help farmers for sustainable rice farming.

A thorough literature review revealed that rice has appreciable potential as an allelopathic crop, however, there is no comprehensive review published earlier in the literature covering multiple allelopathic aspects of this important food crop. In order to bridge the gap of information this review is aimed to provide a deep insight into the allelochemicals in various traits of rice, techniques of investigating allelopathic attributes, modes of action and pathways of allelochemicals production. Moreover, different factors influencing the production of rice allelochemicals along with the significance of rice allelopathy in sustainable agriculture are covered. Understanding these biological phenomena may thus aid in the development of new and novel weed-control tactics while allowing farmers to manage weeds in an environmentally friendly manner.

## 2 Major allelochemicals present in rice

The major allelochemicals in rice are presented in **Table 1**. Allelochemicals in rice have been divided into two categories; phenolic acids belong to one category, whereas terpenoids and flavonoids belong to the other (Li et al., 2020). Allelochemicals from rice exudates include phenolics, fatty acids, benzoxazinoids, and terpenoids. The compounds present in momilactone that inhibits weed growth are a diterpenoid flavone (5,7,4′-trihydroxy-3′,5′- dimethoxyflavone (Tricin)); a cyclohexenone flavone (5,7,4′-trihydroxy-3′,5′- dimethoxy flavone); and a cyclohexanone (3- isopropyl-5-acetoxycyclohex-2-enone) (Iqbal et al., 2019). Chung et al. (2002) noted that the husk extracts of the rice variety, Janganbyeo contained nine compounds, including salicylic acid, which had the highest inhibiting impact on the total seedling length and dry weight of barnyard grass.

**Table 1:** ** **A list of allelochemicals detected from rice plants that inhibit germination and initial growth of weeds.

Source of allelochemicals	Allelopathic compounds	Name of the groups	References
Leaves extracts	Syringaldehyde (4-hydroxy-3,5-imethoxybenzaldehyde)	Benzaldehyde	(Chung et al., 1997; Rimando et al., 2001)
	3β-hydroxy-5α,6α-epoxy-7-megastigmen-9-one and 3-hydroxy-β-ionone	Monoterpene lactone	
Root exudates	Momilactones (A and B)	Diterpenoids	(Kato-Noguchi and Peters, 2013; Kumar et al., 2020)
	Tricin (5,7,4’-trihydroxy- 3’,5’- dimethoxyflavone), 3-isopropyl-5-acetoxy cyclohexene-2-one-1	Flavones	(Khanh et al., 2007 Kong et al., 2019)
	5,4-dihydroxy-3,5-dimethoxy-7-D-β-glucopyranose and 7,4-dihydroxy-3,5-dimethoxy-5-D-β-glucopyranose		
Extract of rice plants	Vanilic acid, syringic acid and p- coumaric acid	Phenolic acids	(Rimando and Duke, 2003)
	Acetic, propionic, butyric,		
	4-hydroxy-3,5- dimethoxybenzaldehyde, 3β-hydroxy-5α,6α-epoxy-7-megastigmen-9-one and 3- hydroxy-β –ionone		(Masum et al., 2016)
Decomposing rice residues	4-hydroxybenzoic acid, 5-methoxysalicyclic acid, 7-oxostigmasterol, 2,4-dimethoxybenzoic acid, 2,5-dihydroxybenzoic acid, 3,4-dimethoxybenzoic acid, 3,5-dihydroxybenzoic acid, 3,5-dihydroxy benzoic acid,	Phenols and fatty acid	(Khang et al., 2016)
	p-hydroxy benzoic acid, p-coumaric acid, ferulic acid, syringic acid and vanillic acid	Phenolics	(Ho et al., 2020)
Hull extracts	Cinnamic acid, coumarin, ergosterol peroxide, p-hydroxycinnamic acid, salicylic acid, vanillic acid	Phenolics	(Chung et al., 2002)

### 2.1 Phenolic acids

Practically all plant species include phenolics, which are naturally occurring chemicals (Macías et al., 2019). Regardless of dose, the phenolic compounds displayed diverse allelopathic actions, with the majority having detrimental effects on the germination of seeds and initial seedling development of barnyard grass (Chung et al., 2002). Phenolic content demonstrated a strong positive relationship with the percent inhibition of barnyard grass root development (Berendji et al., 2008). Another study discovered that the total phenolics content was impacted by methyl jasmonate (MeJA) treatments (Akan and Tuna Gunes, 2021). The grain of the triticale cultivars under investigation included 13 phenolic acids, among which ferulic acid was found to be present in the highest concentration and made up 42-44% of the total phenolic acid content in the grain.

Additionally, the composition of the phenolic acids fraction in the triticale grain of the examined cultivars varied in comparison to that of wheat and rye cultivars because of the high levels of ferulic, di-ferulic, and sinapic acids (Kaszuba et al., 2021). The drying procedure had a substantial impact on the dry matter content, total phenolic content, antioxidant activity, -carotene, flavonoid, and flavonol values (Binici et al., 2021). The phenylpropanoid route leads to the synthesis of phenolic chemicals. Numerous research suggested that phenolics controlled the plants’ defense system response to pathogens such as bacteria, fungi, and viruses. Additionally, this phenolic substance causes significant abiotic pressures including drought and salinity. The structural variety of the phenolic component determines its properties and distribution in various plant species (Das et al., 2020). In plant cells, phenolics are most crucial for appealing to pollinators and discouraging herbivores (Li et al., 2010). Under natural soil conditions, UV light generates a buildup of phenolic compounds in the rhizosphere of rice, which inhibits the growth of weeds (Mahmood et al., 2013).

Phenolic chemicals that are released into the rhizosphere are absorbed by nearby plants together with sap ascent and have an impact on the physiological responses of the receptive plant (Gulzar et al., 2016). Besides, phenolic acids have been linked to a reduction in chlorophyll concentration and thereby a reduction in net photosynthetic rate (Lu et al., 2018). Phenylalanine Ammonia Lyase (P.A.L.) is a crucial enzyme involved in the production of phenolic chemicals (Zhang et al., 2019).

### 2.2 Terpenes

Terpenes, which have five carbon rings and can be added to any group, are also odorous compounds. The chemicals with the greatest potential in the management of insect pests are alkaloids, saponins, phenols, and terpenes, a broad category of secondary plant metabolites (Gajger and Dar, 2021). The genetic engineering of terpenoid-based insect defences is particularly appealing in pest management research. Terpenoids are mostly volatile compounds that serve a range of biological purposes in plants (Croteau et al., 2000). Based on the submergence of compounds, terpenes are categorized as monoterpenes, isoprenoids, and diterpenes, and so on isoprenoid compounds are made up of C5 isoprenoid units (Gershenzon and Croteau, 1994). Several studies have shown that volatile monoterpenes are potent inhibitors of seed germination and root elongation (Singh et al., 2006). Membrane components, reproductive hormones, visual pigments, photo-protective compounds, pheromones, allelochemicals, phytoalexins, and signal molecules are just a few of the many natural functions that terpenoids play (Haig, 2008). In general, the rice plant’s self-defence system and allelopathic potential are known to be heavily dependent on diterpenoids (Kim and Shin, 2003).

#### 2.2.1 Momilactones

Terpenoids, like momilactone B, are commonly considered as possible rice allelochemicals because of their higher inhibitory activity on target weeds at low concentrations (Kato-Noguchi, 2011). Momilactone B is regarded to be the main cause of rice allelopathy (Kato-Noguchi and Peters, 2013). In comparison barnyard grass lettuce and numerous lowland weed species performed much better next to momilactone-scarce rice plants than close to wild-type rice plants (Toyomasu et al., 2014). Momilactones A and B are herbicides that have been patented by Zhao et al. (2018). Momilactones could be used as a source of prospective lead compounds for crop-friendly insecticides to aid in the development of green agriculture.

### 2.4 Essential oils and fatty acids

Basic oils that are lipophilic and aromatics are included in allelochemicals. These are highly volatile compounds produced from polyterpene biosynthesis with an attached fatty acid, which can absorb moisture. All of these compounds are useful for medical purposes, soap manufacturing, linen processing, the food industry, herbal medicines, skin treatments, the paper industry, as herbicides, fertilizer production and biodiesel formation, and antibacterial and antifungal products etc., (Razavi, 2012). The volatile part of myrrh, which primarily consists of sesquiterpenes, has hundreds of metabolites that have been found thus far. Notably, furanosesquiterpenes such furanoelemanes, furanoeudesmanes, and furanogermacranes are the distinctive components of myrrh oil. Sesquiterpenoids, which are significant constituents of volatile oil, have a range of biological properties, including antibacterial, antifungal, and antiparasitic properties that can inhibit the growth of bacteria like A. aureus and Bacillus subtilis as well as the fungus *Aspergillus brasiliensis* and have stronger antioxidative potential (Madia et al., 2021). *Helichrysum italicum* essential oil from France had a greater antioxidant capacity and more significant antibacterial action against the Gram-positive bacteria Staphylococcus aureus and Bacillus subtilis as well as the fungus *Aspergillus brasiliensis* (Mollova et al., 2020).

## 3 Pathways of allelochemicals release

**Figure 1:**
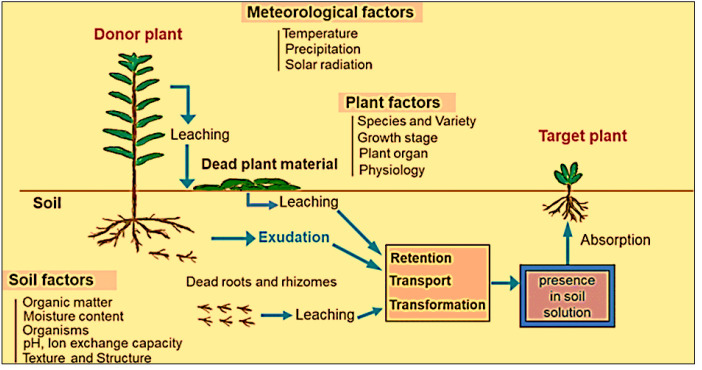
Pathway of allelochemical release from plants (Source: Retrieved and modified from Scavo et al., 2019).

The pathway of the release of allelochemicals in nature is shown in **Figure 1**. Volatile chemicals are released from live plant parts, water-soluble toxins are leached from above-ground portions in response to rain, and others are exuded from below-ground parts (Nega and Gudeta, 2019).

**Figure 1** illustrates the procedure of releasing allelochemicals from the donor plant, then retention, transport or transformation in the soil, and finally how these go to the receiver plant. Therefore, water-soluble allelochemicals from the leaves and plant body surfaces are washed by rain, dew, mist etc. and come to the soil. Some detached leaves on the soil surface are decomposed by the action of microbes and then release allelochemicals which also go to the soil. The living roots and below-ground parts of the donor plants release allelochemicals through exudation. Some dead roots are also decomposed in the soil releasing the allelochemicals. All these four ways are the pathways of releasing allelochemicals from the allelopathic plants. After accumulating in the soil, these may retain in soils, or transform into other compounds before being reached to receptor plant, or it goes directly to the receptor plants.

**Figure 2:**
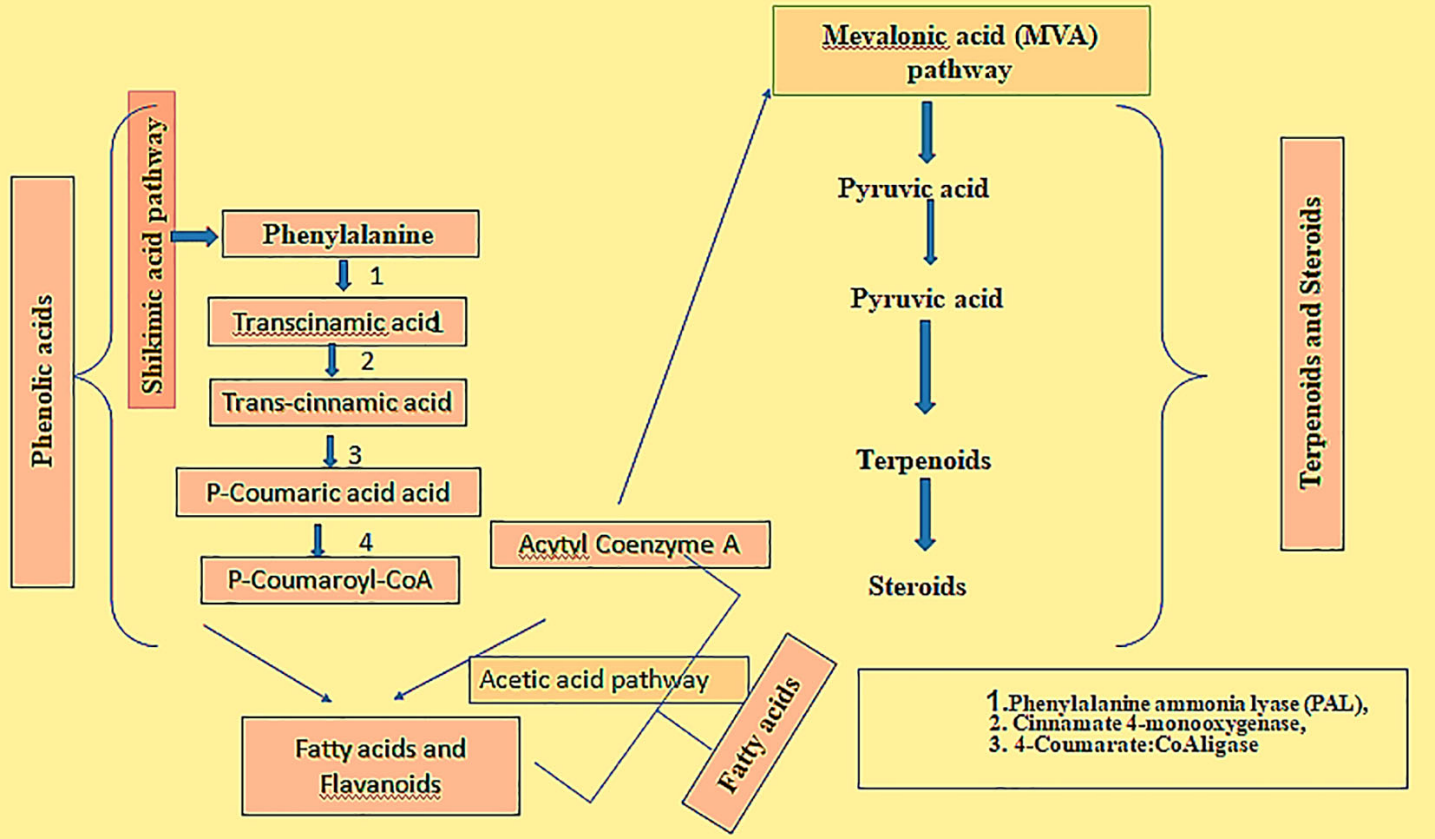
Biosynthesis of major allelochemicals in rice.

During the biosynthesis of allelochemicals in rice, it may follow any of the three routes, i) the shikimic acid, ii) the acetic acid pathway and iii) the mevalonic acid (M.V.A.) pathways (Zhang et al., 2019). The shikimic acid pathway yields phenolic compounds and flavonoids; the acetic acid pathway generates fatty acids and flavonoids; the mevalonic acid (M.V.A.) pathway produces terpenoids and steroids; the shikimic acid and acetic acid pathways combinedly make flavonoids (Buchanan et al., 2015). The biosynthesis of major allelochemicals is presented in **Figure 2**.

## 4 Factors influencing allelochemicals production

The factors which influence the development of allelochemicals in plants can be categorized as a) genetic factors e.g., species of crop, variety of crop, the growth stage of the crop, exponential growing character etc., which are regulated by different genes, and b) environmental factors e.g. Temperature, UV radiation, drought stress, pH level, damages caused by insects, disease pressure, nutrient deficiency etc.

### 4.1 Genetic factors

The allelopathic potential of rice on barnyard grass was lowered by down-regulation of the phenylalanine ammonia-lyase (P.A.L.) gene which decreased phenolic levels and the gene expression of phenolic metabolism-related enzymes. However, abiotic and biotic stressors can cause several genes that affect rice allelochemicals production to become more active. However, abiotic and biotic stressors can cause many genes that affect rice allelochemicals production to become more active (Fang et al., 2013). In a study, it has been observed that the rice variety Makmur, which had more than 40% growth inhibition to barnyard grass could be a valuable gene resource for developing rice cultivars with strong allelopathic potential (Ismail et al., 2007).

Proteomic approaches were utilized to explore the molecular mechanism of crop allelopathy, and four proteins were revealed to be involved in allelochemicals production which are - peroxidase precursor thioredoxin M-type, 3-hydroxy-3-methylglutaryl-coenzyme A reductase 3, phenylalanine ammonia-lyase (P.A.L.), Cinnamate 4-hydroxylase (CA4H). Cinnamate 4-hydroxylase (CA4H) and phenylalanine ammonia-lyase (P.A.L.), are two important enzymes in the phenolic acid production pathway (Fang et al., 2015). Furthermore, these four allelopathy-related proteins are encoded by genes on chromosomes 4, 7, 8, and 12 (He et al., 2005).

The PAL-2-1 gene is one of the key genes involved in allelopathic rice’s ability to limit grass growth (Fang et al., 2013). A quantitative real-time polymerase chain (qRT-PCR) reaction was used to assess genes involved in the biosynthesis of phenolic compounds in rice. The genes recorded in the study are phenylalanine ammonia-lyase (P.A.L.), cinnamate-4-hydroxylase (C4H), ferulic acid 5-hydroxylase (F5H), and caffeic acid O-methyltransferases (C.O.M.T.) (He et al., 2012).

**Figure 3:**
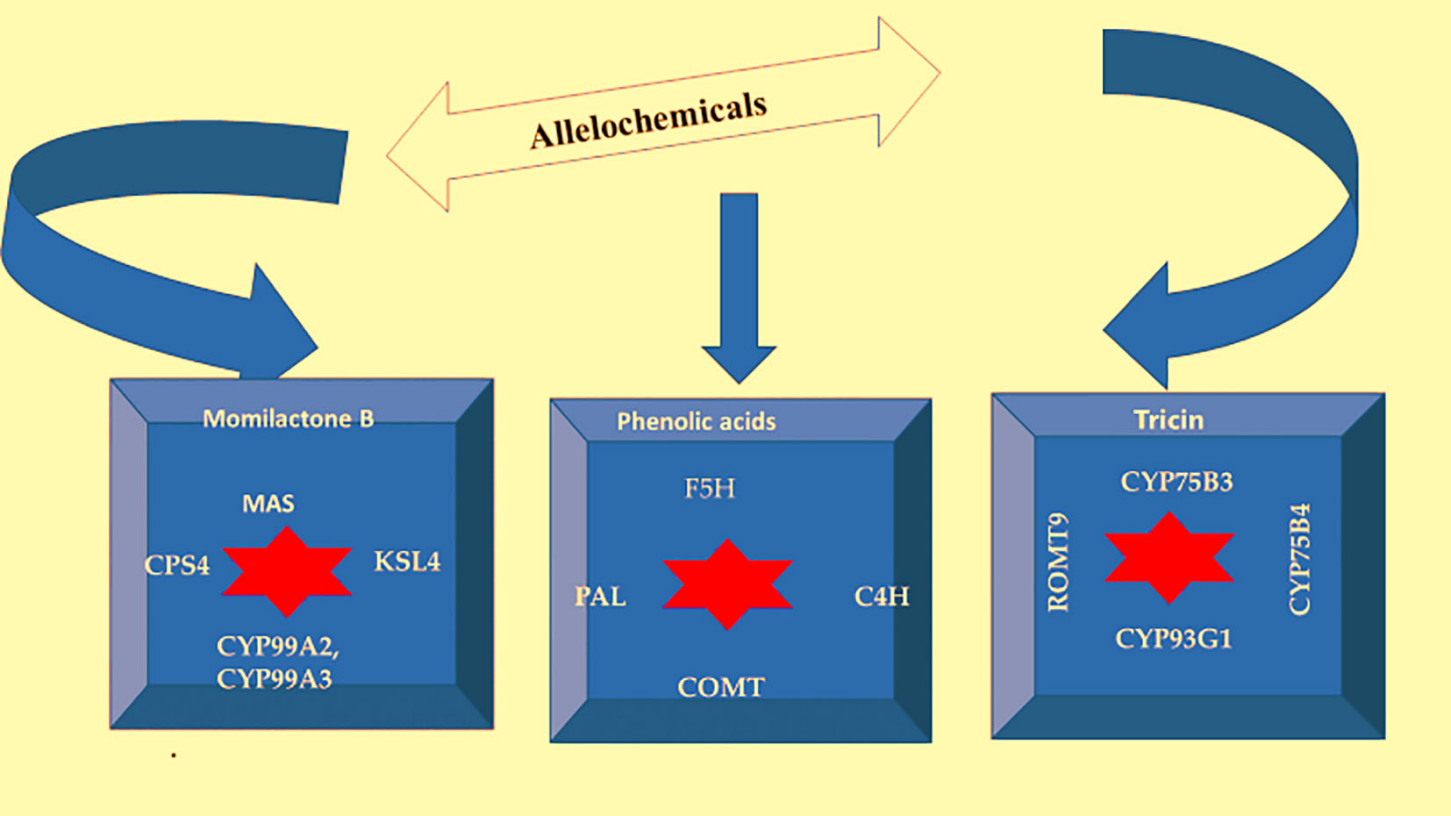
Genes involved in the biosynthesis pathways of momilactone B, phenolics, and tricin [CPS4; gene encoding syn-copalyl diphosphate synthase, KSL4; gene encoding syn-pimara7,15-diene synthase, MAS; gene encoding momilactone A synthase, CYP99A3 and CYP99A2; gene encoding 9-beta-pimara-7,15-diene oxidase, COMT- Caffeic acid *O*-methyltransferases, PAL- phenylalanine ammonia-lyase, Cinnamate-4-hydroxylase (C4H), F5H-Ferulic acid 5-hydroxylase, flavonoid 3’-monooxygenase; CYP75B3, flavonoid 3’-hydroxylase; CYP75B4, flavonoid 3’,5’-*O*-methyltransferase; ROMT9 and CYP93G1; cytochrome flavone synthase II].

The expression of key genes involved in the biosynthesis of momilactone B were OsCPS4, OsKSL4 and OsMAS, CYP99A3 and CYP99A2, from phenolic acids these were COMT, PAL, C4H, F5H, and that of tricin were CYP75B3, CYP75B4, ROMT9 and CYP93G1 (Fang et al., 2013). According to Li et al. ( Li et al., 2020), OsPAL2-1 is one of the most effective genes for reducing rice allelopathy. They also revealed that loliolide and jasmonic acid could induce the expression of genes involved in the biosynthesis of rice allelochemicals as well as the production of these chemicals. According to the findings, the AK103462 gene encodes momilactone A synthase. As a result, this gene has been given the name “*O. sativa* momilactone A synthase” (OsMAS) (Shimura et al., 2007). The genes that take part in the process of biosynthesis for momilactone B, phenolics, and triticin are given below (**Figure 3**).

Momilactone B, a diterpene that is synthesised by diterpene synthase enzymes *via* the methylerythritol phosphate (M.E.P.) route and is an anti-weed allelochemicals is found in rice (Dudareva et al., 2013). However, the Cinnamic acid, produced by PAL from phenylalanine, is the source of plant phenolic compounds. This essential enzyme catalyses the switch from primary (through the shikimate route) to secondary (by the phenylpropanoid pathway) metabolism during the production of phenolic compounds (Sawada et al., 2006). According to the theory, PI312777’s silencing of the PAL gene in allelopathic rice reduces PAL activity, which in turn reduces the release of phenolic chemicals.

### 4.2 Environmental factor

The most important environmental parameters that affect the activity of allelopathy are water and food availability, UV radiation, temperature, and competitive stress (Meiners et al., 2012). Plants in arid regions emit the most volatile compounds, including terpenoids. On the other side, plants in humid climates release phytotoxins that dissolve in water, including phenolics, flavonoids, and alkaloids (Chou, 1999). In a study, it is observed that as temperature and photoperiod were extended, the allelopathic effect was increased at the beginning, then decreased at the 2–3 leaf stage. Therefore, allelopathic substances are produced in greater quantities in an environment driven by stresses. The stressors may control the genes involved in allelochemicals production and release them into the environment (Palanivel et al., 2021).

## 5 Allelochemicals as ecological pesticides

Advanced agricultural activities such as the application of excessive fertilizers, herbicides, fungicides and nematicides etc., have been damaging the soil’s physical and chemical qualities and polluting the soil and water. According to Peterson et al. (Peterson et al., 2018), 51 weed species became common in rice fields as a consequence of extensive pesticide use, which led to the development of herbicide resistance. For the sake of all living things, including humans, natural resources must be conserved and used wisely to preserve the long-term viability of the planet (Chou, 1999).

Allelochemicals found naturally in plants are a promising source of ecological pesticides because they help plants tolerate, suffer, or adapt to insect pest stress. Nowadays, nanotechnology has been contributing to the development of bio-herbicides using allelochemicals. Using allelochemicals to create nanoparticles, nanoemulsions, and nanocapsules and using them as nano-herbicides opens up new possibilities for weed management. The controlled release of nano-herbicides at their smallest effective concentration guarantees the least harmful impact on target field crops and that their bioavailability is maintained (Das et al., 2021). Weed management in farming systems may benefit from the development of allelochemicals-based herbicides. An allelochemicals-based herbicide made of benzothiazine derivatives was patented by Zhao et al. (2019).

## 6 Allelochemicals’ impact on microorganisms and the environment

By producing secondary metabolites as signalling molecules, allelopathy begins communication between plants and microbes in both agricultural and natural habitats. This ultimately affects the growth and development of nearby plants. Allelochemicals produced by these crops control weeds while simultaneously boosting microbial activity underground, demonstrating their allelopathic potential (Jabran et al., 2015). When allelochemicals or allelopathic plants were present, crop development and soil microorganisms had considerable connections. In other words, allelopathic plant species, important mediators, and soil organisms have all been found as significant indicators of how allelopathic interactions between plants will take place (Zeng, 2014).

## 7 Impact of rice allelopathy on weed control

The allelopathic potential of crop plants contributes to cultivars’ capacity to control weeds. Rice allelopathy has thoroughly been investigated, and numerous rice cultivars have been discovered that limited the development of different weed species when cultivated together (Le Thi et al., 2014). Although it is clear that phytotoxins created during the decomposition of rice stubble in the soil are the primary cause of the drop in the yield of the next rice crop, rice plants also have grown successfully for nearly a century with little loss in production. This suggests that rice plants have evolved adaptive strategies to avoid a severe autotoxic response (Chou, 1999). The development of herbicide resistance is a common occurrence as a result of the application of the same pesticide regularly (El-Naby et al., 2019). Therefore, allelopathic rice cultivars should be cultivated to resist these unwanted plants. In a study, it has been observed that the reduction in grain yield of allelopathic rice accessions was roughly 37% in barnyard-grass infested plots, contrary to 60 to 68% in non-allelopathic varieties (Dilday et al., 2001).

Another method of managing weeds is to extract allelochemicals from rice straw (*Oryza sativa* L.) and use them as biocides (Ho et al., 2021). In one study it was exhibited that rice straw extracts suppressed radicle growth in lettuce (*Lactuca sativa*), rice (*O. sativa*), and barnyard grass (Chung et al., 2001). The allelochemicals rho-Coumaric acid dramatically decreased the germination of lettuce seedlings at a dosage of 1 mM. Rho-coumaric acid, on the other hand, was only effective against barnyard grass at concentrations larger than three mM (Rimando et al., 2001).

In the field, rice cultivars with allelopathic activity produced 2–3 times as much root biomass as cultivars with non-allelopathic activity. Rice cultivars with intermediate maturities had a higher proportion of inhibition. Rice cultivars with allelopathic activity had 2-3 times more root biomass than cultivars without allelopathic activity in the field. In comparison to early maturing (50.2%) and late maturing (56.1%), intermediate maturing rice cultivars showed a higher percentage of inhibition (59.3%). Dehulled rice had a stronger inhibitory impact than hulled rice, rice with coloured hulls was inhibited by 55.9%, while colourless hulls were inhibited by 65.4%. Some traditional varieties such as Siam, Jambok, Wangi, and some newer types such as MR77 and MR84 were shown to have considerable allelopathic activity on lettuce and barnyard grass seedlings (Azmi et al., 2000; Mazid et al., 2018). The first allelopathic rice released for commercial application in China in 2009 is Huagan-3 (Kong et al., 2011) and Rondo is an Americal rice cultivar with substantial weed suppressive ability and high yield potential as well (Gealy and Yan, 2012).

## 8 Mode of action of allelochemicals

**Figure 4:**
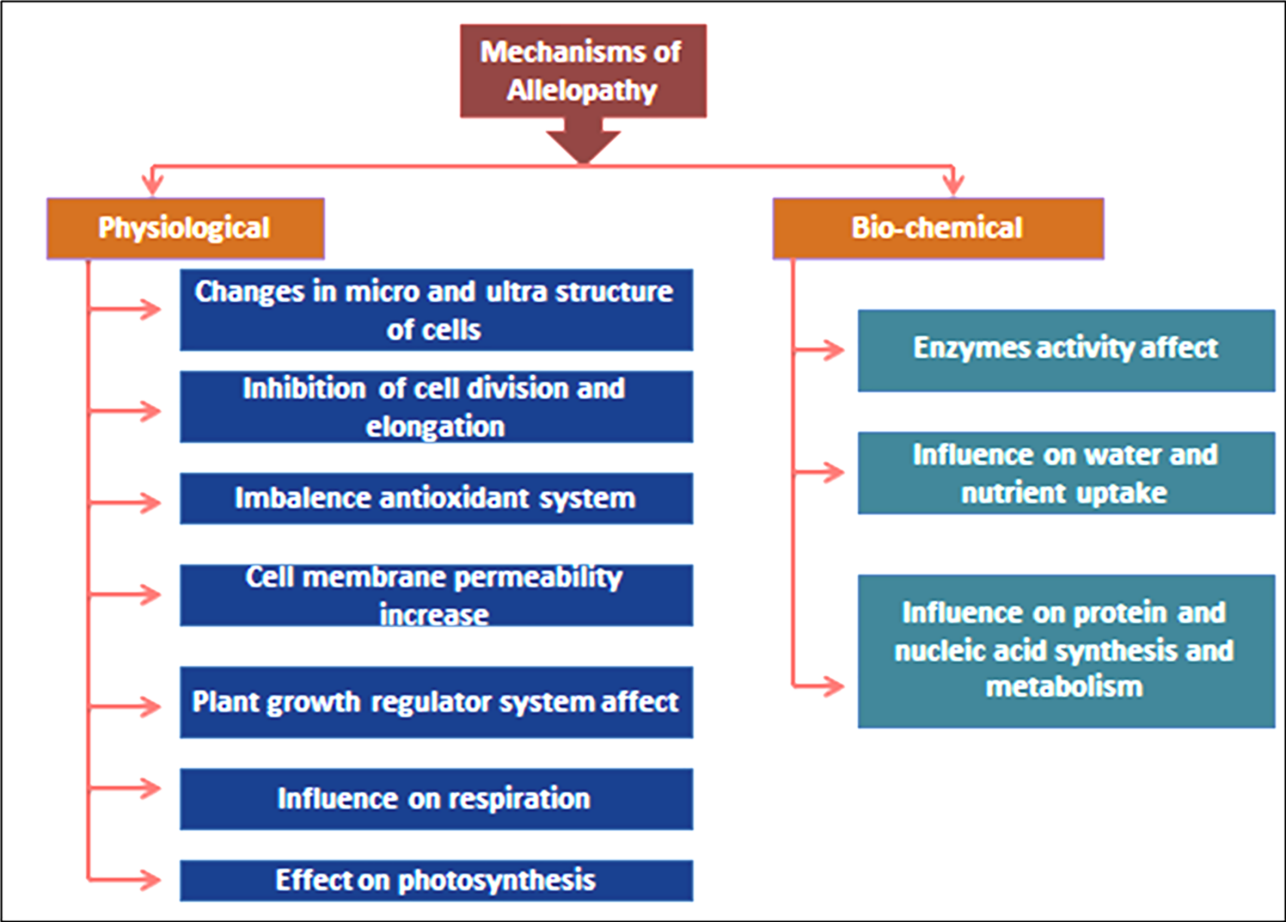
Mechanism of allelopathic effects on receiver plants.

Allelochemicals primarily affect photosynthesis by interfering with photosystem II activity (Wang et al., 2014). Allelochemicals reduce chlorophyll levels, produce free radicals, limit enzyme activity, and alter target plants’ cell membranes and structure, all of which stop weeds from developing (Ghanizadeh et al., 2014). (**Figure 4**) depicts the mode of action of allelochemicals explaining the mechanisms of allelopathy.

### 8.1 Absorption of mineral

Plants’ ion absorption rate can be altered by allelochemicals (Baar et al., 1994). Phenolic acids inhibited both macro and micronutrient absorption (Akemo et al., 2000). The uptake of ammonium (NH_4_^+^) and nitrates (NO_3_^-^) by maize seedlings was shown to be decreased by ferulic acid (250 M), but ammonium intake is far less susceptible to this treatment than NO_3_^-^. Additionally, ferulic acid limits recovery from roots exposed to a low K ammonium nitrate solution increases the first net K^+^ loss, inhibits Cl-uptake, and raises the initial net K^+^loss from roots, all of which cause a favourable net uptake (Bergmark et al., 1992).

### 8.2 Cytology and ultrastructure

Allelochemicals stress triggers the breaking and shrinking of the cells along the epidermis contract, leading to injured margins, a twisted leaf apex, and the development of puff-like structures, and thread-like structures on the surface of the leaf. The allelochemicals have been identified to limit mitosis in a variety of ways in plant roots (Mushtaq et al., 2019).

### 8.3 Phytohormones

Allelochemicals influence the generation of two plant growth hormones such as IAA (Indole acetic acid) and GA (gibberellins), which control cell size in plants. The IAA-oxidase inactivates IAA synthesis, and various allelochemicals stimulate IAA-oxidase (Chou and Kuo, 1986). Allelopathic stress in plants increases the production of ethylene and ABA (abscisic acid) as well (Bogatek et al., 2005). In barnyard grass, an aqueous extract of rice was found to drastically increase IAA oxidase activity and decrease IAA levels, harming the plant’s growth regulatory system and preventing seedling growth (Wenxiong et al., 2001).

### 8.4 Membrane permeability

The influence of various organic substances is mediated through changes in membrane permeability (Galindo et al., 1999). By blocking plasma-membrane, H^+^-ATPase, mitochondrion, and chloroplast electron transport chains, modifying protein metabolism, and producing oxidative stress, allelochemicals produce cellular abnormalities that are reflected in morphological changes and reduced the growth rates in plants (Gomes et al., 2017). It also results in the loss of membrane integrity which leads to cellular injury. Moreover, it causes increased conductivity levels resulting in the disruption of membrane integrity. A decrease in membrane permeability could be due to the peroxidation of polyunsaturated fatty acids in the biomembranes (Singh et al., 2006). Numerous studies have demonstrated that allelochemicals greatly reduce the activity of antioxidant enzymes and raise free radical levels, which causes increased membrane lipid peroxidation and membrane potential alteration, which reduces the ability of activated oxygen to scavenge and harms the entire membrane system of plants (Wenxiong et al., 2001).

### 8.5 Photosynthesis

Allelochemicals such as benzoic and cinnamic acid lowered the amount of chlorophyll in soybeans, preventing photosynthesis (Baziramakenga et al., 1994). They function as electron acceptors, electron uncouples, energy-exchange inhibitors, or a combination of these, ultimately slowing down the pace of photosynthetic reactions (Batish et al., 2001).

### 8.6 Respiration

Allelochemicals can fortify or repress respiration, both of which are injurious to the vitality of the energy-producing system (Batish et al., 2001). The allelochemicals, cinnamic acid, and α-pinene decreased electron transport to the alternative pathways in the respiratory mechanism in soybean (Peñuelas et al., 1996).

### 8.7 Protein synthesis

Studies that monitored the incorporation of radio-labelled C14 sugars or amino acids into proteins found that allelochemicals inhibit protein synthesis (Bertin et al., 2007). In addition to blocking amino acid transport, allelochemicals can also prevent amino acid absorption. This prevents protein synthesis, which hinders cell growth. The integrity of DNA and RNA can be impacted by all phenolic acids (Abenavoli et al., 2003).

### 8.8 Enzyme activity

Allelochemicals inhibit the activity of particular enzymes in plants (Muscolo et al., 2001). Allelochemicals from tex-mex tobacco (*Nicotiana plumbaginifolia)*, for example, increased the activity of catalase (CAT) and superoxide dismutase (SOD) in sunflower seedlings (Singh et al., 2015).

### 8.9 Conducting tissue

Tracheids, xylem arteries, xylem parenchyma, and xylem fibers are the principal elements of plant growth. Allelochemicals obstructed nutrition absorption in plants through these plant tissues (Gniazdowska & Bogatek, 2005).

### 8.10 Plant-water balance

Allelochemicals also influence osmotic potential and leaf water potential, which results in an imbalanced shoot turgor pressure (Sheteawi & Tawfik, 2007). After being treated with ferulic acid, p-coumaric acid, and extracts from several allelopathic weeds, grain sorghum (Sorghum bicolour (L.) Moench.) seedlings grown under summer glasshouse conditions showed improved leaf diffusive resistance and decreased water potential (Einhellig et al., 1985).

## 9 Genetic and molecular research in allelopathy

The molecular research on allelopathy started many days ago. In 1996, the International Rice Research Institute (IRRI) started a research program on genetic monitoring of allelopathy and genes implicated in chromosomal allelopathy in rice, as well as an analysis of quantitative properties of allelopathy as a primary tool of genetic research (Courtois and Olofsdotter, 1998). These breeding programs for creating genetic variability in rice varieties might be a viable strategy for improving paddy in weed management (Kong et al., 2011). On the other hand, Jansen and Nap (2001) used DNA markers to map gene expression and to classify allelopathic-linked epistatic quantitative trait loci (QTL).

Researchers have made significant progress in discovering genes that code for momilactones in rice (Xu et al., 2004). Lee et al. (2005) identified QTLs on chromosomes 1, 2, 3, 4, 5, 8, 9, and 12 that regulate the allelopathic effects of rice against *E. crus-galli*. The most common allelopathic QTLs were found on chromosomes 1 and 5, accounting for 36.5% of overall phenotypic variance. Two methods for producing more allelopathic crops have been proposed modulating gene expression associated with allelochemicals production and inserting genes into non-allelopathic crops for generating allelochemicals (Duke et al., 2001). Allelopathic traits related to various chromosomal regions have also been observed in other crops, such as wheat (Olofsdotter, 1998).

The rice varieties, MR439 and MR164 among 10 simple-sequence repeats (SSR) markers investigated were the rice genotypes with substantial allelopathic activity (El-Denary et al. (2016). The varieties showed polymorphic DNA patterns, and the primer RM164 exhibited a band with a molecular weight of 296bp in all genotypes and had strong allelopathic activity. In another research, SSR227 was found useful in selecting allelopathic rice using the marker-assisted selection method (El-Naby et al., 2019). It was considered the most superior and prospective genetic material for effective weed control and producing the highest yield (El-Denary et al., 2016). DNA marker-assisted selection has recently been proved as a tool for finding QTLs responsible for allelochemicals production in a range of crops, including rice and rapeseed (Shehzad and Okuno, 2020). In a QTL analysis study, Chung et al. (2020) discovered a QTL on chromosome 8 that limits shoot growth and overall length. All this information emphasizes genetic upgrading of allelopathic features as a necessary pre-requisite for using rice allelopathy for sustainable weed management in rice fields (Chen et al., 2008). Jansen and Nap (2001) noted that there was no considerable association between the root morphology and their allelopathic potential, suggesting that allelopathy in rice was under genetic control irrespective of root morphology. From the results of another study on chromatin immunoprecipitation and HiSeq data, it was confirmed that the gene OsMYB57 transcriptionally controlled a mitogen-activated protein kinase, OsMAPK11 and this OsMAPK11 was interrelated with OsPAL2, which is the gene responsible for rice allelopathy. Therefore, the gene OsMYB57 improved rice allelopathy, which allowed genetic engineering for improving weed control in rice. Furthermore, increasing OsMYB57 expression in rice using the transcription activator VP64 resulted in stronger inhibitory effects against barnyard grass (Fang et al., 2020). Molecular mechanisms of allelopathy, soil dynamics in crop weed competition, genetic mapping of QTLs, and knowledge of the mechanism of action of allelochemicals are all related to the development of allelopathic crop types (Palanivel et al., 2021).

## 10 Screening techniques for determining the allelopathic potential of rice

Different methods and techniques have been used by researchers to detect the allelopathic potential in plants. Rice bioassay is a simple and efficient tool for determining the allelopathic potential of rice cultivars during the earliest stages of allelopathic research (Khanh et al., 2007). The stair-step method, hydroponic culture test, relay-seeding technique, agar medium test, cluster analysis using HPLC, and water extract method all are examples of techniques to screen rice for allelopathic potential (Maqbool et al., 2013). A short description of different techniques with their references is given below in **Table 2**.

**Table 2:** The screening methods usually used to detect allelopathic potential in rice.

Sl. No	Name of method	Short description	Receiver plant	References
1.	Relay seeding technique	• First rice seeds are sprouted in Petridish with moistened filter paper, after 11 days, the receiver weed seeds are placed near to germinating rice seeds, which release allelochemicals in the growth medium. Effects are measured by estimating loss in root, shoot and biomass of the weed in comparison to control.	Barnyard grass	Mazid et al., 2018
2.	Sandwich method	• Between two layers of agar solution, some crushed rice leaf, stem, husk, and other rice debris are kept uniformly and 20 test weed seeds are put on agar medium close to smashed rice seeds. After ten days, different seedling traits are measured as mentioned above.	Barnyard grass	Yoshiharu Fujii et al., 2003
3.	Leaf, root and hull extracts bioassay	• Leaf and hull extracts of rice are used in a growth medium in the laboratory (e.g. petridish) where the test weed seeds are placed for germination. After 10 days the seedling parameters as mentioned early are measured. Sometimes root extracts of the rice varieties are collected and tested in the laboratory for phytotoxicity. This technique is usually done first to identify allelopathic potential and then to identify the most active chemical responsible for allelopathic action by column fractioning of different allelochemicals followed by testing with weed species.	Barnyard grass	Chung et al., 1997
4.	Pot culture bioassay with plant extracts	• A greenhouse pot is filled with soil along with an appropriate amount of N, P, K, Ca, Mg, Fe etc. Seven pre-germinated receiver test weeds are sown in the pot. An amount of 250 ml of 10% aqueous solution of rice leaf is added to the weed seedlings at a 3-leaf stage in the pot. Ten days after the addition of extracts the weeds are harvested for measurement of weed parameters.	Barnyard grass	Masum et al., 2016
5.	Equal compartment agar bioassay	• An agar solution (0.3%) is autoclaved and then solidified in a beaker. Six pre-germinated rice seeds are placed in one half of the agar cycle. After seven days, the other portion of the agar surface is covered with 10 pre-germinated re-receiver weed seeds. The beaker is crossed in the middle and the middle of a piece of whiteboard is placed, keeping the board 1 cm above the surface. Again after 10 days, the seedling parameters are assessed.	Lettuce, Cress, Radish,*E. coli*,Jungle rice	Masum et al., 2016
6.	Plant box method	• This technique involves transplanting rice seedlings into a root-separating cellulose tube occupied with 0.5% water agar medium, which is then positioned in the middle of one side of a sterilized square plastic box that has been filled with 1 lit of 0.5% water agar solution. A highly engineered holding structure holds the dialysis tube. Receive weed seeds are sterilized and then arranged in rows close to the dialysis tube on the agar surface. The allelochemicals released by the rice plants move to weed seedlings and affect the growth of the weed.	Lettuce andBarnyard grass	Azmi et al., 2000
7.	Field screening following additive technique	• Rice seedlings are raised in seedling boxes and 45 days old seedlings are transplanted in the well-prepared field in five 30 cm rows with a 15 cm spacing between rows (30 x 15 per 3.3 m^2^). Forty days old seedlings of receiver test weed (barnyard grass) raised in seedling boxes, are planted in five rows across the rice rows two weeks after they are planted. Recommended N, P, K, Ca, Mg, Fe etc. are applied and no herbicide is sprayed. The allelopathic effects of rice on the weed are evaluated after 67 days of rice planting based on per cent loss in weed parameters.	Barnyard grass	Ahn et al., 2005
8.	24-well plate bioassay	• Twelve-day-old rice seedlings are placed in the 10 cm apart holes in a Styrofoam float which is placed in a 24 L pail that is filled with hydroponic solution. The float allows the rice roots to submerge in the hydroponic solution. The solution is replaced after every 2.5 days and the pH is maintained at 5.5. After one month of growth, the roots are separated, dried and powdered. The powdered root mass is mixed with methanol (50:50) to prepare root extract. The extract is dissolved in acetone to make 10% strength and is tested for selected weed species.* Five weed seeds are placed on the filter paper which is set at the bottom of the well containing 20 µLof the extract. The plate is sealed with parafilm and placed in a growth chamber at 25 ^0^C and 16 hrs photoperiod for four days. After that, the measurement of the shoot and root lengths is done and the per cent reduction in comparison to control is estimated.	Barnyard grass	Rimando et al., 2001
9.	Root exudation bioassay	• Hydroponically grown 15 days old 51 rice seedlings are bound to an urethrane foam which is placed at the top of 100 mL paper cups containing 100 mL of sterile deionized water. The cups are aerated continuously with a vinyl chloride tube connected to an air pump and are replaced every week. The root exudate solutions are collected from the cups and are filtered using appropriate filter papers. The seeds of test weeds are placed in a petridish for germination and the exudation solutions are added to the germinating medium. The measurement of allelopathic effects is done by estimating the percent loss in root and shoot parameters as mentioned early in comparison to control.	Barnyard grass	Tawaraya et al., 2018

## 11 Significance of allelopathy in sustainable agriculture

Sustainable agriculture involves optimizing farm resources while relying on the basic minimum of purchased inputs. It also aims to reduce the impact of agricultural activities outside of farm limits. The main goal of sustainable agriculture is to decrease pesticide use and the need to focus on developing alternative weed control strategies that are low-cost, environmentally safe, and long-lasting (Sathishkumar et al., 2020). To attain the goal of sustainable agriculture, plant breeding, soil fertility and tillage, crop protection, and cropping methods must all be thoroughly explored and utilized (Chou, 1999). Substantial alternative technologies are required to protect our planet from being polluted and to support the increased global population in recent decades (Wezel et al., 2014). Even though agricultural yield varies widely worldwide, herbicides have become a common approach for weed control in most places (Peterson et al., 2018). However, the herbicide use should be in a way to reduce the detrimental effects on the crop and the agroecosystem. For example, the benzothiazine derivative is an allelochemicals-based herbicide that is eco-sustainable (Kong et al., 2019).

## 12 Conclusion

Weeds infestation in rice fields is a great concern for the people behind rice. Although a good number of chemical herbicides can control the weeds effectively in rice fields; which lead to environmental degradation. Rice allelopathy in this regard is one of the best options for environmentally friendly weed management tactics in rice. The development of allelochemicals-based bio-herbicides and the development of allelopathic rice varieties through breeding techniques might be very useful for weed control in sustainable agricultural systems. At long last, using allelopathic rice variety or introgression of the gene responsible for an allelopathic trait may open a new window for the farmers to get the desired output and help to develop smart agriculture.

## Author contributions

Conceptualization, ASJ. Methodology, AJ, MR, MU and LH. Validation, ASJ, MR, MU, and FR. Formal analysis, FR. SM, and HB. Investigation, FR and HB. Resources, FR and AJ. Data curation, FR. Writing—original draft preparation. Editing, RK, ASJ, MR, MU, LH, AC, OY, AH, and BYR. Visualization, FR, MA, and MI. Supervision, AJ, MR, and MU. Project administration, AJ, and FR. All authors have read and agreed to the final version of the manuscript. All authors contributed to the article and approved the submitted version.
